# Exploring the Nature of the Antimicrobial Metabolites Produced by *Paenibacillus ehimensis* Soil Isolate MZ921932 Using a Metagenomic Nanopore Sequencing Coupled with LC-Mass Analysis

**DOI:** 10.3390/antibiotics11010012

**Published:** 2021-12-22

**Authors:** Mohamed A. Eltokhy, Bishoy T. Saad, Wafaa N. Eltayeb, Ibrahim S. Yahia, Khaled M. Aboshanab, Mohamed S. E. Ashour

**Affiliations:** 1 Department of Microbiology, Faculty of Pharmacy, Misr International University (MIU), Cairo 19648, Egypt; mohammad.ashraf@miuegypt.edu.eg (M.A.E.); wafaa.eltayeb@miuegypt.edu.eg (W.N.E.); 2 Department of Bioinformatics, HITS Solutions Co., Cairo 11765, Egypt; bishoyth@hitssolutions.com; 3 Laboratory of Nano-Smart Materials for Science and Technology (LNSMST), Department of Physics, Faculty of Science, King Khalid University, P.O. Box 9004, Abha 61413, Saudi Arabia; ihussein@kku.edu.sa; 4 Research Center for Advanced Materials Science (RCAMS), King Khalid University, P.O. Box 9004, Abha 61413, Saudi Arabia; 5 Nanoscience Laboratory for Environmental and Biomedical Applications (NLEBA), Semiconductor Lab., Department of Physics, Faculty of Education, Ain Shams University, Roxy, Cairo 11757, Egypt; 6 Department of Microbiology and Immunology, Faculty of Pharmacy, Ain Shams University, Organization of African Unity Str., Cairo 11566, Egypt; 7 Department of Microbiology and Immunology, Faculty of Pharmacy, Al-Azhar University, Cairo 11651, Egypt; seifashour@hotmail.com

**Keywords:** *Paenibacillus ehimensis*, LC/MS, metagenomics, β-lactone, petrobactin, tridecaptin, locillomycin, polymyxin, terpene, polyketide

## Abstract

The continuous emergence of multidrug-resistant (MDR) pathogens poses a global threat to public health. Accordingly, global efforts are continuously conducted to find new approaches to infection control by rapidly discovering antibiotics, particularly those that retain activities against MDR pathogens. In this study, metagenomic nanopore sequence analysis coupled with spectroscopic methods has been conducted for rapid exploring of the various active metabolites produced by *Paenibacillus ehimensis* soil isolate. Preliminary soil screening resulted in selection of a Gram-positive isolate identified via 16S ribosomal RNA gene sequencing as *Paenibacillus ehimensis* MZ921932. The isolate showed a broad range of activity against MDR Gram-positive, Gram-negative, and *Candida* spp. A metagenomics sequence analysis of the soil sample harboring *Paenibacillus ehimensis* isolate MZ921932 (NCBI GenBank accession PRJNA785410) revealed the presence of conserved biosynthetic gene clusters of petrobactin, tridecaptin, locillomycin (β-lactone), polymyxin, and macrobrevin (polyketides). The liquid chromatography/mass (LC/MS) analysis of the *Paenibacillus ehimensis* metabolites confirmed the presence of petrobactin, locillomycin, and macrobrevin. In conclusion, *Paenibacillus ehimensis* isolate MZ921932 is a promising rich source for broad spectrum antimicrobial metabolites. The metagenomic nanopore sequence analysis was a rapid, easy, and efficient method for the preliminary detection of the nature of the expected active metabolites. LC/MS spectral analysis was employed for further confirmation of the nature of the respective active metabolites.

## 1. Introduction

The increased emergence of antibiotic resistance among microbes poses a worldwide threat. Hence, there is an urgent need for discovery of new antimicrobials. Soil microbial diversity is a very rich source of microorganisms that produce secondary metabolites possessing antimicrobial activity. Selman Waksman, a leader in antibiotic discovery, organized a study that has led to the discovery of streptomycin [1]. The later antibiotic was isolated from *Actinomyces griseus* [2]. Another example was the isolation of gramicidin from *Bacillus brevis* [3]. During the golden age of antibiotic discovery between 1940 and 1950, novel antibiotics were easily discovered by random conventional screening methods. However, random screening methods proved to be time consuming and tedious; as an example, daptomycin was obtained by screening one to ten million *Actinomycetes* isolates. Herein, new advanced methods for discovery of novel antibiotics are a global necessity to discover novel antimicrobial agents to combat multidrug-resistant microorganisms [4].

Through the years of the 1960s and 1980s, several antibiotic classes were developed and used, but later, the lucrative progress of new antimicrobials has been hindered. On the other side, continuous evolution of bacterial resistance developed, resulting in serious incurable bacterial infections imposing a public health threat. Further progress in this area requires multidisciplinary studies including structural biology, enzymology, bioinformatics, metabolomics, transcriptomics, proteomics, and advances in mass spectral analysis, and it is believed that this will lead to the “second golden age for antibiotics” and natural product detection [5].

The facultative anaerobic *Paenibacillus* is a motile, endospore-forming bacteria found in various habitats. *Paenibacillus* was previously identified within the genus of *Bacillus* [6]. However, after the development of molecular identification techniques, later in 1993, it was identified as a separate genus and now is under the family *Paenibacillaceae*. The different species are Gram-variable, as they can be either Gram-positive or Gram-negative [7].

Antimicrobial production varies between species due to the diversity in antimicrobial-encoding genes [8]. The produced antimicrobials by *Paenibacillus* include peptides, volatile organic compounds, and enzymes [9]. The produced enzyme shows a potent antifungal activity. These enzymes include proteases, cellulases, glucanases, and chitinases [9]. *Paenibacillus ehimensis* KWN38 bioactive metabolites can break the structure of hypha and stop the growth of *Rhizoctonia solani*, *Phytophthora capsic,* and *Fusarium oxysporum* [9,10]. The chitinase produced by some *Paenibacillus* spp. is stable with other fungicides, suggesting a potential combination [11].

The produced antimicrobial peptides are of two types: non-ribosomally synthesized peptides and ribosomally-synthesized bacteriocins. It is known that *Paenibacillus* produces two out of the three classes of bacteriocins [9]. Several species such as *Paenibacillus kobensis* and *Paenibacillus alvei* are known to produce polymyxins [9].

A study conducted on *Paenibacillus polymyxa* (P13) showed antimicrobial activity against *Lactobacillus* species [12]. The extracted antimicrobial was effective against several Gram-positive and Gram-negative bacteria, showing bacteriocin-like properties, and was stable when heated up to 90 °C. It was also stable at acidic pH, but not at alkaline pH, and it was insensitive to chelators and organic solvents [12].

In the second decade of sequencing, metagenomics analysis was introduced. Metagenomics analysis identifies all DNA present in a given community [13]. Metagenomics made it possible to discover antimicrobials from natural sources [14] as bioinformatics analysis and systematic characterization made it possible to explore the hidden biosynthetic gene clusters [15].

The technological advancement in genome mining and sequencing rejuvenated the examination of under-explored soil environments [16]. The different microbial communities and co-existence in a specific environment influence the evolution of bacteria and genetic expressions. As discovered, bacterial symbionts of marine invertebrates produce several novel natural products. Most bioactive polyketides were isolated from a marine bacterial symbiont called *Candidatus Entotheonella* sp. with the marine sponge *Theonella swinhoei* [17]. The *Clostridium* genus was not affiliated with any form of antimicrobial production until genomic data were studied and investigated. Studying *Clostridium cellulolyticum* under laboratory conditions revealed no antibiotic production. However, studying conditions that mimic the soil environment by adding aqueous soil extract to the fermentation process prompted the antimicrobial production for closthioamide [18].

The importance of chromatographic methods was highlighted by a study of secondary metabolites extracted from four isolates habituated in industrial wastewater. The purpose of the study was to discover novel antimicrobial agents. The characterization of the targeted elute was performed using reversed phase high-performance column chromatography (RP-HPLC) [19]. Another study characterized antimicrobial compounds obtained from screened fungi that have bioactivity against clinical isolates. The functional group of the compound was characterized using gas chromatography–mass spectrometry (GC–MS) [20].

From the previous studies, we decided to rediscover antimicrobial metabolites production by *Paenibacillus* genus, isolated from different Egyptian soil samples. The study started by applying conventional preliminary soil phenotypic screening methods. Then, a metagenomic nanopore sequence analysis of the soil sample harboring the promising *Paenibacillus* genus was carried out to identify the conserved biosynthetic gene clusters of the produced active metabolites. Afterward, LC–mass spectroscopy was conducted to confirm the nature of the respective antimicrobial metabolites.

## 2. Results

### 2.1. Screening of the Antimicrobial Activities of the Recovered Bacterial Isolates

Table 1, Table 2 and Table 3 showed the results of the preliminary screening for the antimicrobial activities of the three recovered bacterial isolates, coded SP1, SP2, and SP3, that phenotypically and biochemically belonged to *Paenibacillus* spp. Each isolate was recovered from a different soil sample. The isolate SP1 showed inhibition against all the tested standard and MDR Gram-positive, Gram-negative, and *Candida* isolates. Therefore, the soil sample harboring the isolate SP1 was selected for a metagenomic analysis and LC–mass spectral analysis of the produced metabolites.

### 2.2. Molecular Identification 

The16S ribosomal RNA gene sequencing of the isolate coded SP1 revealed 99% identity to NCBI reference sequence of *Paenibacillus ehimensis* strain IFO 15659. Therefore, the isolate coded SP1 was identified as *Paenibacillus ehimensis* isolate MZ921932. The 16S ribosomal RNA gene sequence was analyzed and deposited in the National Center of Biotechnology Information (NCBI) GenBank under nucleotide accession number MZ921932. 

### 2.3. The Antimicrobial Activities of the Extracted Metabolite(s) of Paenibacillus ehimensis Isolate MZ921932

Dichloromethane extract showed zones of inhibition against MDR bacteria than the ethyl acetate extract. However, the ethyl acetate extract showed bigger zones of inhibition against tested *Candida* spp. than the Dichloromethane extract, as shown in Table 4.

### 2.4. Metagenomics Analysis of the Soil Sample

Results revealed a maximum read count of 600,000 reads, and the mean sequence length was 1000 bp. Sequence length (bp) ranged from 100–10,000 bp. FastQ quality score per base showed good quality (Phred score range between 10 and <25). Percentage N count (ambiguous) was zero, and there were no significant duplicate reads.

Percent abundance of the bacterial phylum present in the soil showed that the most abundant phylum was *Achromobacter.* The organism with the most abundance was *Pseudarthrobacter* sp. NIBRBAC000502770, representing 17% of the sample, followed by *Arthrobacter phenanthrenivorans* sphe3 of 10%. *Paenibacillus ehimensis* belongs to Firmicutes phylum, which was present in the soil sample with around 15% abundance. Metagenomics sequences were deposited in the NCBI GenBank under accession number PRJNA785410 (https://www.ncbi.nlm.nih.gov/sra/PRJNA785410, accessed on 5 December 2021) and Figure S1 (Supplementary) illustrates the soil microbial diversity.

### 2.5. Identification of Secondary Metabolite(s) Gene Clusters Paenibacillus ehimensis Isolate MZ921932

#### 2.5.1. Siderophore Petrobactin

**Figure 1:**
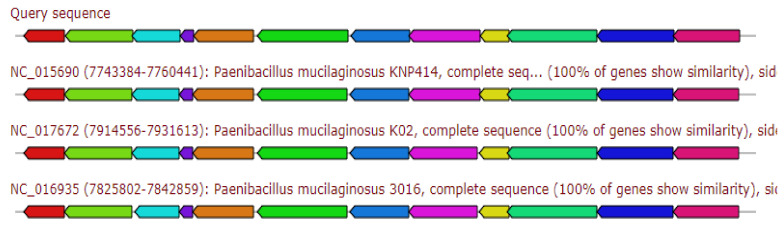
Gene arrangement of siderophore petrobactin gene cluster homologous to query sequence of *Paenibacillus ehimensis* isolate MZ921932 to query sequence. Putative biosynthetic genes presented in red, additional biosynthetic genes in orange, transport-related genes in blue, regulation-related genes in green, resistance genes in pink, and TTA codon in dark pink; grey represent other genes.

The resulted gene cluster showed 83% similarity to the siderophore Petrobactin biosynthetic gene cluster (Figure 1).

#### 2.5.2. Traditional (Multi-)Modular Non-Ribosomal Peptide Synthases

**Figure 2:**
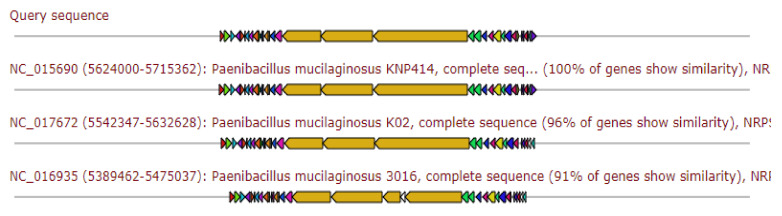
Gene arrangement of Tridecaptin gene cluster homologous to query sequence of *Paenibacillus ehimensis* isolate MZ921932 to query sequence. Putative biosynthetic genes presented in red, transport-related genes in blue, and regulation-related genes in green.

Gene cluster showed 80% similarity to the tridecaptin biosynthetic gene cluster, as displayed in Figure 2.

#### 2.5.3. Hybrid Region: Beta-Lactone Containing Protease Inhibitor and Non-Ribosomal Peptide Fragment

**Figure 3:**
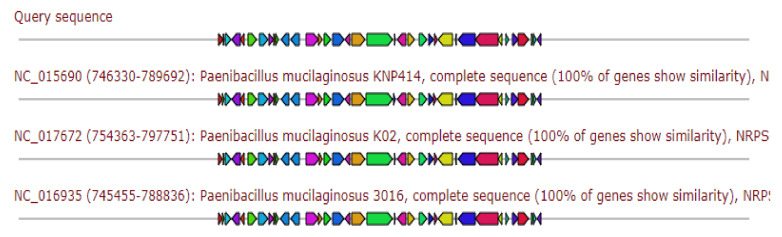
Gene arrangement of β-lactone gene cluster homologous to query sequence of *Paenibacillus ehimensis* isolate MZ921932 to query sequence. Putative biosynthetic genes presented in red, additional biosynthetic genes in orange, transport-related genes in blue, regulation-related genes in green, resistance genes in pink, and TTA codon in dark pink; grey represents other genes.

The gene cluster gave 80% similarity to the Locillomycin biosynthetic gene cluster, as depicted in Figure 3.

#### 2.5.4. Traditional (Multi-)Modular Non-Ribosomal Peptide Synthases

**Figure 4:**
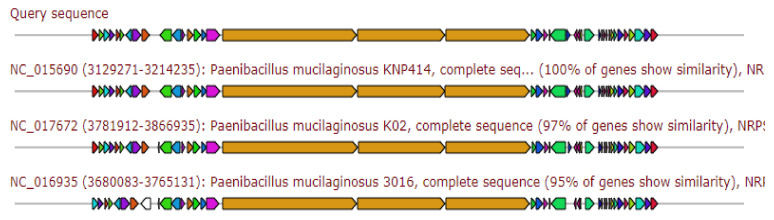
Gene arrangement of Locillomycin gene cluster homologous to query sequence of *Paenibacillus ehimensis* isolate MZ921932 to query sequence. Putative biosynthetic genes presented in red, additional biosynthetic genes in orange, transport-related genes in blue, regulation-related genes in green, resistance genes in pink, and TTA codon in dark pink; grey represents other genes.

Gene cluster gave 42% similarity to Locillomycin biosynthetic gene cluster as depicted in Figure 4.

#### 2.5.5. Hybrid Region: Thioamide-Containing Non-Ribosomal Peptide and Traditional (Multi-)Modular Non-Ribosomal Peptide Synthases

**Figure 5:**
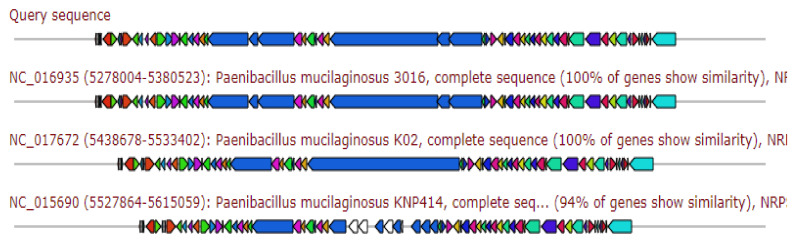
Gene arrangement of Polymyxin gene cluster homologous to query sequence of *Paenibacillus ehimensis* isolate MZ921932 to query sequence. Putative biosynthetic genes presented in red, additional biosynthetic genes in orange, transport-related genes in blue, regulation-related genes in green, resistance genes in pink, and TTA codon in dark pink; grey represents other genes.

The resulted gene cluster revealed 40% similarity to the biosynthetic gene cluster of Polymyxin A and Polymyxin B, 40% similar to the gene cluster producing Colistin/Colistin B (Figure 5).

#### 2.5.6. Terpene

**Figure 6:**
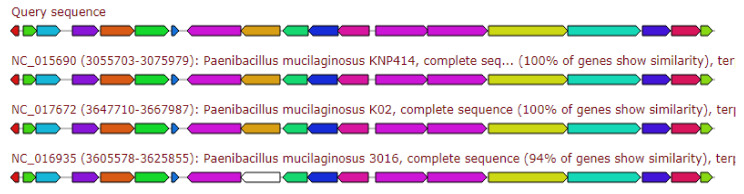
Gene arrangement of Carotenoid terpene gene cluster homologous to query sequence of *Paenibacillus ehimensis* isolate MZ921932 to query sequence. Putative biosynthetic genes presented in red, additional biosynthetic genes in orange, transport-related genes in blue, regulation-related genes in green, resistance genes in pink, and TTA codon in dark pink; grey represents other genes.

The resulted gene cluster showed 40% similarity to biosynthetic gene cluster of the produced Carotenoid (Figure 6).

#### 2.5.7. Trans-AT Polyketide 

**Figure 7:**
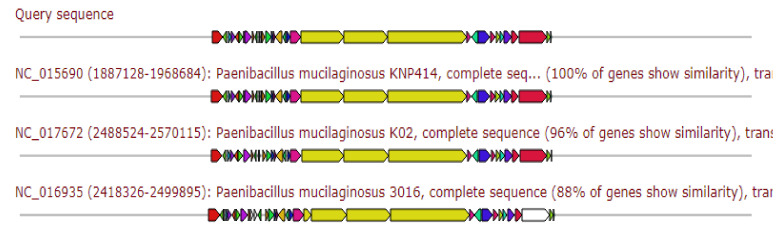
Gene arrangement of polyketide gene cluster homologous to query sequence of *Paenibacillus ehimensis* isolate MZ921932 to query sequence. Putative biosynthetic genes presented in red, additional biosynthetic genes in orange, transport-related genes in blue, regulation-related genes in green, resistance genes in pink, and TTA codon in dark pink; grey represents other genes.

Gene cluster was 20% similar to the gene cluster producing the Difficidin, 20% similar to the gene cluster producing the Macrobrevin, and 13% similar to the gene cluster producing the Sorangicin (Figure 7).

#### 2.5.8. Hybrid Region: Thioamide-Containing Non-Ribosomal Peptide and Traditional (Multi-)Modular Non-Ribosomal Peptide Synthases

**Figure 8:**
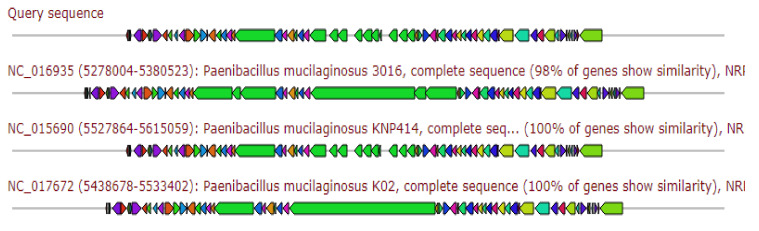
Gene arrangement of polymyxin gene cluster homologous to query sequence of *Paenibacillus ehimensis* isolate MZ921932 to query sequence. Putative biosynthetic genes presented in red, transport-related genes in blue, regulation-related genes in green, resistance genes in pink, and TTA codon in dark pink; grey represents other genes.

The gene cluster showed 60% similarity to the gene cluster of the producing Paenibacterin, 40% similarity to the gene cluster producing the Polymyxin B, the gene cluster producing Tridecaptin, and the gene cluster producing colistin A/colistin B, and 20% similarity to the gene cluster producing Fengycin (Figure 8).

### 2.6. Characterization of the Antimicrobial Metabolite(s)

#### 2.6.1. TLC Analysis

The spots of the separated compounds were observed under a UV lamp at 365 nm (fluorescence) and 254 nm (absorbance). In case of dichloromethane extract, TLC using solvent system ethyl acetate: methanol (9:1) showed four separated spots, while solvent system ethyl acetate: methylene chloride (9:1) and dichloromethane: ethanol (6.5:3.5) showed no significant separation. Ethyl acetate extract, TLC using solvent system ethyl acetate: methanol (9:1) showed two separated spots, while TLC using solvent system ethyl acetate: methylene chloride (9:1) and dichloromethane: ethanol (6.5:3.5) showed no significant separation (Figure S2). 

#### 2.6.2. LC/MS Analysis

##### Ethyl Acetate Extract

**Figure 9:**
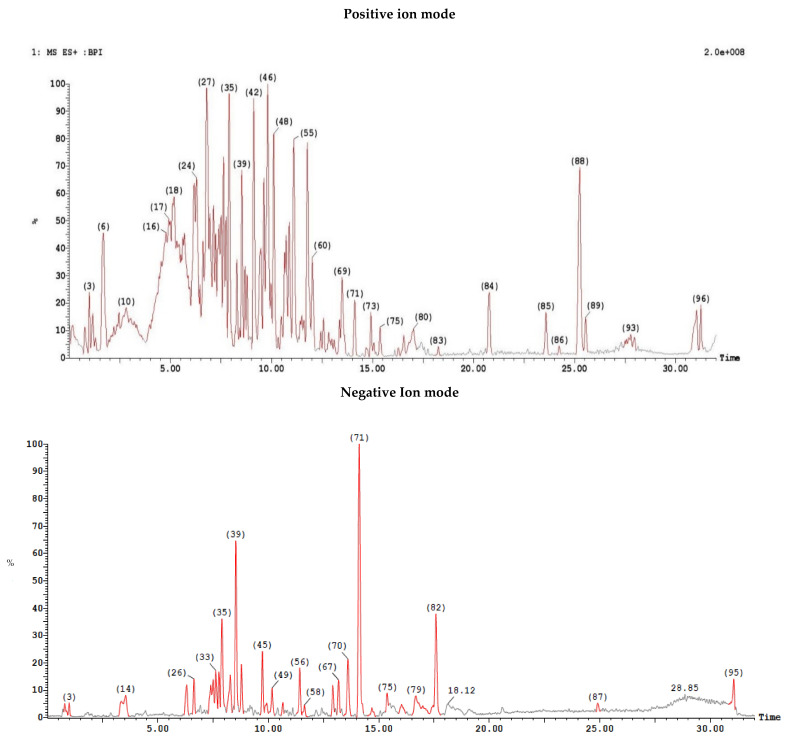
A cell-free ethyl acetate extract run on both ESI-MS positive and negative ion mode for antibiotic detection from the isolate *Paenibacillus ehimensis* isolate MZ921932 using XEVO TQD triple quadrupole instrument.

LC/MS carried on cell-free ethyl acetate extract generated 95 peaks in negative ion mode and 96 peaks in positive ion mode, illustrated in Figure 9, and each peak had variable masses (Figure 9).

**Figure 10:**
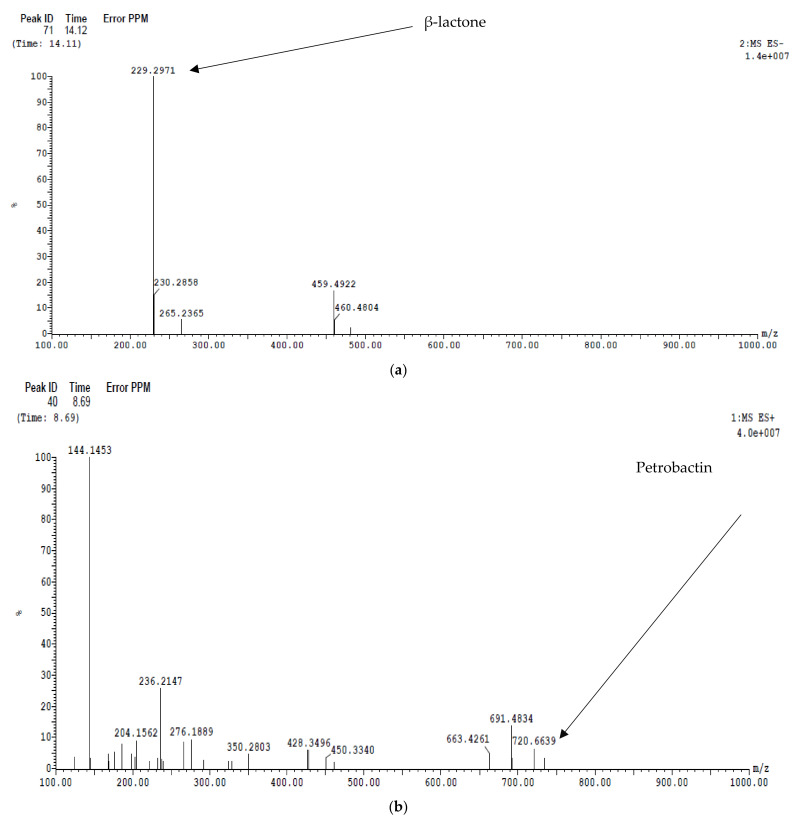

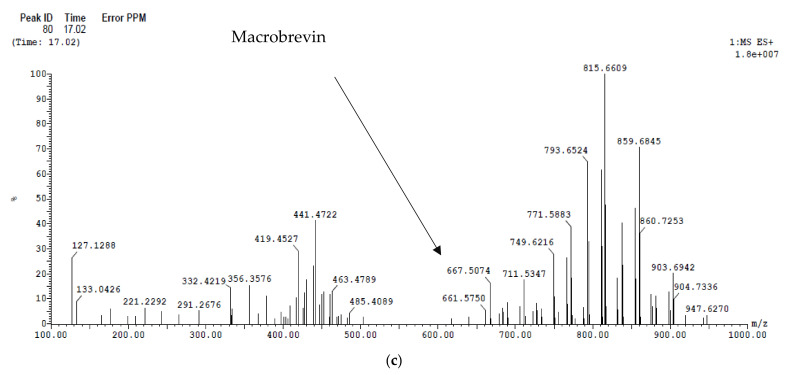
LC/MS analysis of a cell-free ethyl acetate extract of *Paenibacillus ehimensis* isolate MZ921932 detection of (**a**) β-lactone, (**b**) petrobactin, and (**c**) macrobrevin.

The mass spectra were analyzed for the detection of the secondary metabolite of *Paenibacillus ehimensis* isolate MZ921932, as illustrated in Figure 10. (a) Locillomycin (β-lactone) was detected at peak 71 at time 14.12 min with *m*/*z* 229.29. (b) Petrobactin was detected at peak 40 at time 8.69 min with *m*/*z* 720.663. Macrobrevin was detected at peak 80 at time 17.02 min with *m*/*z* 667.507.

##### Dichloromethane Extract

**Figure 11:**
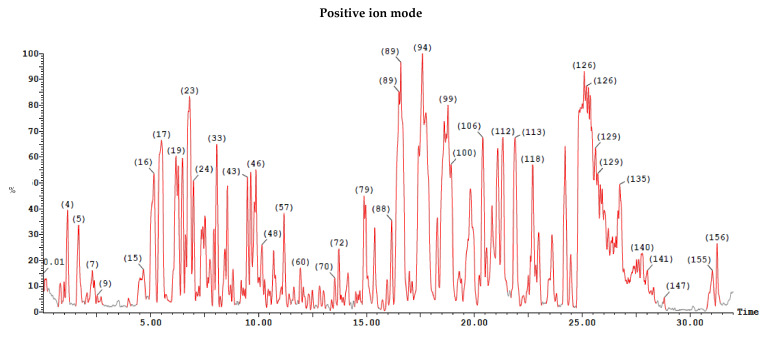

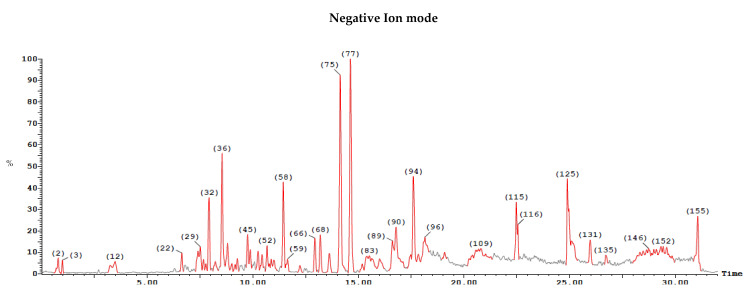
A cell-free dichloromethane extract run on both ESI-MS positive and negative ion mode for antibiotic detection from the isolate *Paenibacillus ehimensis* isolate MZ921932 using XEVO TQD triple quadrupole instrument.

LC/MS was carried on cell-free dichloromethane extract generated 155 peaks in negative ion mode and 156 peaks in positive ion mode, illustrated in Figure 11.

**Figure 12:**
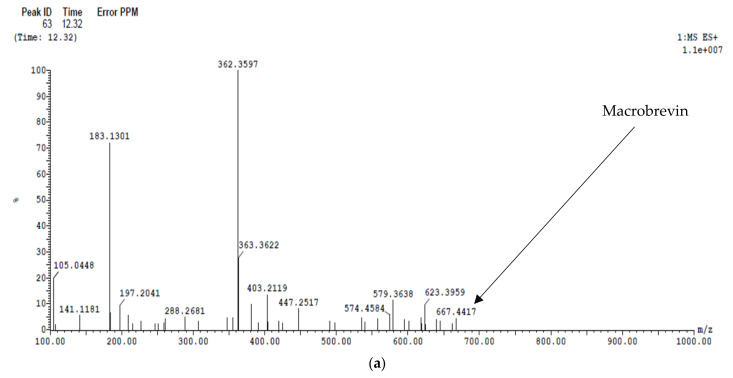

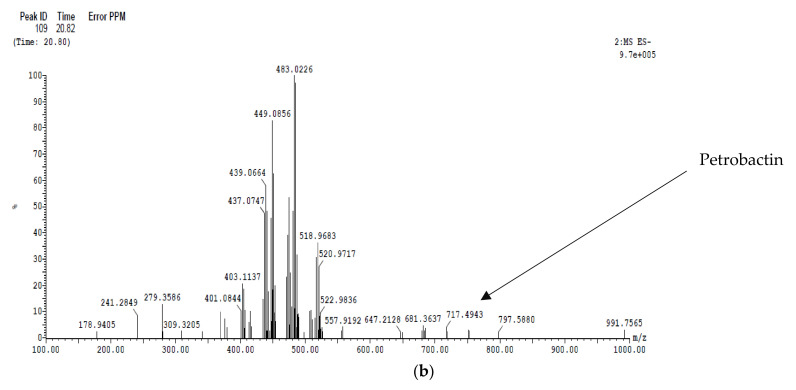
LC/MS analysis of a cell-free dichloromethane extract of *Paenibacillus ehimensis* isolate MZ921932. Detection of (**a**) macrobrevin and (**b**) petrobactin.

The mass spectra of the dichloromethane extract were analyzed for the detection of the secondary metabolite of *Paenibacillus ehimensis* isolate MZ921932, as illustrated in Figure 12. (a) Macrobrevin was detected at peak 63 at time 12.32 min with *m*/*z* 667.44. (b) Petrobactin was detected at peak 109 at time 20.82 min with *m*/*z* 717.49. 

## 3. Discussion

Natural sources have always been a refuge when it comes to the antibiotic discovery. Soil, a good habitat for antimicrobial production, is an important origin for antimicrobial discovery as *Streptomyces* species isolated from the soil proved to be the source of the streptomycin antibiotic [16,21]. Previous literature highlighted the potential of antimicrobial discovery from different species of *Paenibacillus* [9] as well as from different soil organisms [22,23]. In the present study, *Paenibacillus ehimensis* was isolated from the soil collected from Jabal Al-twailat, Dahab, Egypt. The isolate exhibited a broad spectrum of antimicrobial activities as it showed activity against the standard and MDR Gram-positive and negative bacteria as well as fungi test organisms. Other studies showed a different antimicrobial spectrum [24]. A study emphasized on the antifungal and antibacterial activity exhibited by *Paenibacillus polymyxa* PKB1 [24]. The isolate bioactivity was assayed against *Leptosphaeria maculans* for the detection of antifungal activity and against *E. coli* for antibacterial activity [24]. Another study that isolated *Paenibacillus tyrfis* from a Malaysian swamp exhibited broad spectrum antimicrobial activity [25]. The isolated organism was tested against *P. aeruginosa* ATCC 10145, *E. coli* ATCC 25922, *C. albicans*, MRSA 700699, methicillin-sensitive *S. aureus* (MSSA) ATCC 29213, and vancomycin-resistant *Enterococcus* (VRE) ATCC 700802 and showed activity against the listed organisms [25]. *Paenibacillus elgii*, an isolate from soil sample located in Tianmu Mountain National Nature Reserve (Hangzhou, China), showed antifungal activity and antibacterial activity against both Gram-positive and Gram-negative bacteria [26]. Another research on Argentina isolated *Paenibacillus polymyxa* (P13) from fermented sausage identified an antimicrobial named polyxin, of proteinaceous nature, exhibited activity against a wide array of Gram-positive and Gram-negative bacteria [12]. *Paenibacillus polymyxa* NRRL B-30507 has proven to produce bacteriocin, which is proteinaceous in nature, and showed antimicrobial activity against food-borne bacteria, such as *Campylobacter jejuni* [27,28]. Another strain of *Paenibacillus polymyxa* isolated from food exhibited broad spectrum antimicrobial activity [29]. It showed activity against *E. coli* 0157:H7, *Listeria monocytogenes* (three strains, including the processing-resistant OSY-8578), *S. enterica* (four strains, including the multidrug-resistant DT109 and FM 12501-51), *Salmonella enterica*, *Yersinia enterocolitica*, *S. aureus*, and *Bacillus cereus*, but showed no activity against fungi. Its activity was attributed to a previously known antimicrobial polymyxin E1 (active against Gram-negative bacteria), a novel antibiotic 2,983-Da compound (active against Gram-positive bacteria), and designated as paenibacillin after purification [29]. Paenibacillin was tested and proven effective against *Clostridium sporogenes*, *Bacillus* spp., *Lactobacillus* spp., *Leuconostoc mesenteroides*, *Lactococcus lactis*, *Listeria* spp., *Pediococcus cerevisiae*, *Streptococcus agalactiae*, and *S. aureus* [29]. A bacteriocin-like peptide was produced from *Paenibacillus ehimensis* isolated from a soil sample in India [30]. Its activity was assessed against *S. aureus* (MTCC 1430), *Listeria monocytogenes* (MTCC 839), *Bacillus subtilis* (MTCC 121), *Vibrio cholerae* (MTCC 3904), *P. aeruginosa* (MTCC 1934), *E. coli* (MTCC 1610), *Saccharomyces cerevisiae* (MTCC 170), *C. albicans* (MTCC 1637), *Asperigillus niger* (MTCC 281), *Fusarium oxysporum* (MTCC 2773), and MRSA [30]. The isolate exerted activity against both Gram-positive and Gram-negative bacteria but showed no inhibition against fungi [30].

Previous studies conducted on the methods of fermentation and extraction of the selected isolate *Paenibacillus ehimensis* isolate MZ921932 proved to be a contributing factor that affects the antimicrobial production and spectrum of activity [31,32]. The study conducted on the antimicrobial production by *Paenibacillus polymyxa* PKB1 was performed by fermenting in a glucose-starch-CaCO3 (GSC) medium followed by methanol extraction [24]. The isolated *Paenibacillus tyrfis* from a Malaysian swamp was maintained on tryptic soy agar (TSA) at 30 °C for 3–4 days, and extraction was carried out [25]. The isolate *Paenibacillus elgii* from China was fermented in the fermentation medium (0.3% sucrose, 1% peptone, 0.5% NaCl, 0.3% soluble starch, pH (7.0–7.2)) at 30 °C for 24 h [26]. Extraction of the bioactive compounds was performed by n-butanol: water (1:1) twice [26]. The antimicrobial polyxin was obtained from *Paenibacillus polymyxa* (P13) after growth in a brain heart infusion (BHI) medium at 30 °C for 72 h [12]. *Paenibacillus polymyxa* NRRL B-30507 producing bacteriocin was grown in modified Kugler’s broth medium supplemented with tryptophan, alanine, and glucose at 32 °C for 40 h and, after centrifugation, the protein contents were precipitated [28]. The strain of *Paenibacillus polymyxa* isolated from food was grown on tryptic soy agar containing 0.6% yeast extract (TSAYE) at 30 °C for 24 h. After centrifugation, the inoculated medium was allowed to pass through a resin, and the adsorbed antimicrobial within the resin was suspended in ethanol for antimicrobial extraction [29]. *Paenibacillus ehimensis* isolate from India was grown in cell-free fermented nutrient broth to produce a bacteriocin-like compound for 48 h at 30 °C [30]. Isolation of the active compound was performed by incubating the fermented media with Diaion HP20 resin [30].

*Paenibacillus polymyxa* PKB1 was analyzed first using HPLC, and eluted fractions were then assayed for antimicrobial activity [24]. LC/MS was then performed to analyze bioactive peaks and after purification matrix-assisted laser desorption ionization–Fourier transform ion cyclotron resonance–mass spectrometry (MALDI–FTICR–MS) for confirmation of the peptide sequence. The last confirmation was performed using GC/MS to confirm the identity of each amino acid in the peptide sequence [24]. The isolated *Paenibacillus tyrfis* dried extract was eluted in HPLC to acquire the active fraction, and partial purification was performed using preparative HPLC [25]. The collected extract from *Paenibacillus elgii* isolated from China was analyzed using MCI GEL column chromatography [26]. The eluted active fraction was then purified using a preparative HPLC system. The active fraction was then dried, and amino acid analysis was performed using the advanced Marfey’s method with LC/MS. The analysis concluded that two novel active antimicrobials were produced related to the pelgipeptin family, named Pelgipeptin A and B [26]. The molecular weight of polymyxin produced from *Paenibacillus polymyxa* (P13) was studied by gel filtration on a Sephadex, and then, fractions were assayed for antimicrobial bioactivity [12]. Bacteriocin produced from *Paenibacillus polymyxa* NRRL B-30507 was purified using gel filtration method and ion exchange chromatography, followed by antimicrobial detection in eluted fractions [28]. Bacteriocins were analyzed using gel electrophoresis and isoelectrofocusing. The gel strips were then analyzed for microbial inhibition [28]. Further detection of bacteriocin was performed by amino acid sequencing using Edman degradation, and its molecular mass was determined using matrix-assisted laser desorption [28]. The extract isolated from *Paenibacillus polymyxa* isolated from food was purified using HPLC, and the antimicrobial fraction was collected and analyzed using LC/MS. The amino acid sequence of the antimicrobial was analyzed using MALDI-TOF MS, NMR, and MS/MS and was later identified as paenibacillin [29]. Antimicrobial fractions produced from *Paenibacillus ehimensis* isolated from India were purified using gel filtration chromatography and further processed using HPLC. The final product was analyzed using circular dichroism (CD), NMR, PEGylation, and MALDI [30].

Utilizing metagenomics analysis for antimicrobial discovery is a promising approach [14]. In the current study, a soil sample was collected from Jabal Al-twailat, Dahab, Egypt for analysis. The collected soil sample revealed only three isolates exhibiting different antimicrobial activities. The most promising one was the identified isolate *Paenibacillus ehimensis* isolate MZ921932, which showed broad spectrum antimicrobial activity as well as antifungal activity, which was uncommon to be associated with the genus *Paenibacillus*. Several studies analyzed soil sample using metagenomics. Analysis of the biosynthetic gene clusters (BGC) of *Actinobacteria*, *Acidobacteriota*, *Verrucomicrobiota,* and *Gemmatimonadota* found in the Mars Oasis in the Southern Maritime Antarctic showed that they were able to produce various antimicrobial metabolites [33]. A deep sequencing using Illumina HiSeq 2500 platform of Mantag Mangrove forests soil demonstrated that *Proteobacteria* (≅55%) was the most abundant, followed by *Firmicutes* (≅11%) and *Bacteroidetes* (≅7%). *Actinobacteria*, Chloroflexi, *Cyanobacteria,* and *Planctomycetes* were also present (≅3–5% each) [34]. The different microbial habitats show an opportunity for antimicrobial discovery. 

AntiSMASH analysis was performed on the soil sample collected in this study for the detection of secondary metabolite gene clusters. The correlation of LC/MS analysis with the identified secondary metabolite gene clusters was proven to be an efficient method for the identification of bacterial metabolites. The gene clusters of *Paenibacillus ehimensis* isolate MZ921932 were targeted as they showed a promising activity against both MDR Gram-positive and Gram-negative bacteria as well as pathogenic *Candida* spp. Similarity between the query sequences and other pre-identified secondary metabolite gene clusters suggested several metabolites. There was a genetic similarity of 83% to a siderophore called petrobactin. Petrobactin is a low-molecular-mass iron (3)-chelating compound. Siderophore antibiotic conjugate is an approach to overcome cellular barriers; in the case of petrobactin, it is essential for iron uptake in bacteria, in which a petrobactin antibiotic conjugate can act as a Trojan horse for the antibacterial activity [35,36]. Tridecaptin showed its potential against Gram-negative bacteria with low cytotoxicity as it blocks ATP synthesis in bacteria through a lipid-II-binding motif [37,38]. A genetic similarity of 100% to β-lactones was also found. β-lactones are natural products that have been described to have potent antifungal and antibacterial activity on human cancer cell lines. Chemically, they are four-membered heterocycles with high ring-strain, electrophilicity, and reactivity [39]. A gene cluster similarity of 42% to the locillomycin producing gene cluster was also found [39]. Locillomycin was found to show moderate activity against MRSA and a significant antiviral activity with great potential for clinical use as it has low cytotoxicity [40]. A 40% gene similarity to Polymyxin A and Polymyxin B (Colistin A and Colistin B) producing genes was concluded. It is widely known that colistin is used to treat serious Gram-negative infections [41]. A gene cluster with 40% similarity to carotenoid (terpene) production of terpenes was found. Terpenes were previously associated with antimicrobial activity [42,43]. A gene cluster showed similarity to different types of polyketides, difficidin, macrobrevin, and sorangicin, with 20%, 20%, and 13% similarity, respectively. Several polyketides have several antimicrobial activities, such as rythromycins and rifamycins with different mechanisms of actions [44]. Thus, a hybrid region was also found in the gene cluster analysis with similarity to Paenibacterin, Polymyxin B, Tridecaptin, Colistin A/Colistin B, and Fengycin-producing gene clusters. The exhibited antimicrobial activity of the isolate *Paenibacillus ehimensis* MZ921932 is an outcome of the collective action of the identified secondary metabolites. 

Compared to other studies, an isolated strain of *Paenibacillus alvei*, a strain related to this group, has great potential to produce antimicrobial metabolites [44]. The identified BGCs were performed using the whole genome sequencing by Illumina platform. The identified BGCs with 100% similarity were bicornutinA1/A2, paenibactin, polymyxin, paenibacterin, icosalide A/B, and anabaenopeptin NZ857 [45]. Colistin A/B, paenibacterin, tridecaptin, polymyxin B, pellasoren, marthiapeptid, and pelgipeptin genes were also identified with lower similarity. Other NRPS and hybrid cluster with less than 25% similarity or total novelty suggested new antimicrobial compounds [45]. A study that collected a soil sample from rhizosphere and tomatoes resulted in isolation of *Bacillus* and *Paenibacillus* with antagonistic activity [46]. Alongside known BGCs, such as surfactin, bacillibactin, and fengycin, novel BGCs were identified from the isolates [46]. *Paenibacillus polymyxa* E681 isolate from South Korea sequence revealed several BGCs including NRPS, PKSs, and bacteriocin [47]. Eleven BGCs were identification using the antiSMASH database [47].

## 4. Materials and Methods

### 4.1. Isolation and Characterization

A collection of soil samples was gathered from different localities in Egypt. For the purpose of decreasing the microbial count, the soil samples were air dried for one week in shade followed by heating for one hour at 80 °C in a hot air oven for further physical treatment [48,49]. Prepared suspensions of soil samples (1 g per 9 mL saline) were vortexed at 400 rpm for 4 min [50,51]. A series of 10-fold serial dilutions in six tubes were prepared. For every dilution, 1 mL was spread on the surface of Starch Casein Agar (SCA) [49]. Preliminary screening was performed against various bacterial isolates to determine its inhibitory effect at the end of a 7-day incubation period. Preliminary identification of soil bacteria was performed through microscopical analysis and variable biochemical tests [52,53].

### 4.2. Preliminary Screening

Bacterial isolates recovered from different soil samples were selected according to their antimicrobial activities against standard *E. coli* ATCC 25922 and clinical isolates of three vancomycin resistance *S. aureus* (VRSA1, VRSA2, and VSRA3), three *Staphylococcus (S.) epidermidis* (SE1, SE2, and SE3), two MDR *E. coli* (EC1 and EC2), three multidrug-resistant (MDR) *K. pneumoniae* (KP1, KP2, and KP3), three *Candia* (*C.) auris* (CS1, CS2, and CS3), and three *C*. *albicans* (CA1, CA2, and CA3), and the clinical isolates were discharged from the Central Microbiology Lab of Ain Shams Hospital. Table 5 represents the antimicrobial resistance patterns of the respective clinical isolates. All recovered isolates from the soil were inoculated on Mueller Hinton agar (MHA) and the test organisms were inoculated perpendicularly to the isolate [54,55]. The plates were then incubated for 24 h at 37 °C. The formation of an inhibition zone around the tested organisms indicated the antimicrobial activity of the respective isolate [54,56]. 

### 4.3. 16 S Ribosomal RNA Gene

According to results of preliminary antimicrobial screening, 16S ribosomal RNA of the most promising was sequenced and analyzed by GATC Biotech Co., Germany through Sigma Scientific Services Co., Egypt. The provided contig of the 16S ribosomal RNA was aligned and blasted in GenBank database using Basic Local Alignment Search Tool (BLAST, https://blast.ncbi.nlm.nih.gov/Blast.cgi, accessed on 5 December 2021) provided by NCBI. The result was presented as percentage homology between the query sequence and those provided by the database. Multiple Sequence Comparison by Log-Expectation (MUSCLE, https://www.ebi.ac.uk/Tools/msa/muscle/, accessed on 5 December 2021) was used to determine the alignment of the 16S ribosomal RNA sequence against the database to retrieve phylogenetic tree. Phylogenetic tree was inferred via likelihood method with a bootstrap analysis (1000 replicates). The 16S ribosomal RNA sequence was deposited in the NCBI GenBank.

### 4.4. Production of the Antimicrobial Metabolite(s) in Shake Flasks

#### Seed Culture Preparation and Growth Conditions

The seed culture was prepared by transferring a loopful of fresh culture of the promising bacterial isolate(s) into 50 mL starch casein broth and incubated at 200 rpm at 35 °C for 24 h. About one mL of the culture was centrifuged for 5 min at 16,000 rpm using a micro centrifuge tube, washed twice with 1 mL sterile saline, and used to inoculate the production flasks (100 mL of casein starch broth X 20 flasks). These flasks were incubated in a shaking incubator (150 rpm) for 7–10 days at 35 °C [57]. Centrifugation at 10,000 rpm for 10 min was performed for separation of the biomass. The filtrate was then passed through a 0.45 μm membrane filter (Merck, Darmstadt, Germany) for separation of the bacterial cells from the culture medium.

### 4.5. Purification of the Antimicrobial Metabolite(s)

Extraction was performed sequentially using the solvent extraction method. The solvents used were ethyl acetate and dichloromethane. Equal volumes of ethyl acetate were added first to the filtrate in a separating funnel [57]. The mixture was agitated for 2 h at 10 min intervals and left over night for complete separation. The upper organic layer was collected and stored at 4 °C for further analysis. The previous steps were repeated for dichloromethane [57]. The organic layers from ethyl acetate and dichloromethane were dried using a rotary evaporator (Buchi R205, Flawil, Switzerland) at 45 °C [56,58,59].

### 4.6. In Vitro Testing of the Antimicrobial Activities of the Extracted Metabolite(s)

The ethyl acetate and dichloromethane crude extracts were both dissolved in DMSO [54]. The antimicrobial testing was performed using both extracts and the filtrate left after extraction with both solvents using well diffusion method [56]. The two organic extracts were tested against eight organisms (*S. aureus* ATCC 25293, VRSA2, EC1, EC2, KP1, KP2, *C. albicans* ATCC 10231, and CA1). A negative control well was filled with DMSO [56].

### 4.7. Metagenomics Analysis of the Soil Samples

#### 4.7.1. DNA Extraction and Quantification

Qiagen DNeasy power-soil kit (Cat. no. 12888-50 Qiagen, Hilden, Germany) was used for DNA extraction as per manufacturer protocol. After DNA extraction quantification of DNA was measured by Qubit fluorometer ver. 4.0 to guarantee there is enough pure genomic material before the sequencing run, 400 ng/7 μL (55 ng/μL), as mentioned by Oxford nanopore manual. Metagenomics were performed at HITS Solutions Co (Bioinformatics Department, Cairo, Egypt, http://www.hitssolutions.com/ accessed on 5 December 2021) [15]. 

#### 4.7.2. Library Construction

Library construction was performed using a Rapid Sequencing Kit (Oxford Nanopore Technologies, Oxford, UK, Cat. # SQK-RAD004). Before loading on the flow cell, a total of 34 μL of sequencing Buffer and 25.5 μL of loading Beads were added to 12 μL of the DNA libraries and 4.5 μL nuclease free water. After that, priming and loading onto FLO-MIN106 flow cell was performed [15].

#### 4.7.3. Sequencing and Data Analysis

Sequencing was run on MinION™ (Oxford Nanopore Technologies) for 12 h, which generates 3.03M reads with N50 equals 9.29K. Base calling was performed in real time during sequencing by the Guppy software. which generates FAST5 and FASTq files, and reads below Q7 were eliminated. A centrifuge was used to classify sequencing reads to a taxonomic identifier, as previously reported [60]. Results were visualized using re-centrifuged [15].

#### 4.7.4. Genome Sequencing Aligning and Analysis

AntiSMASH version 2 (Antibiotics and Secondary Metabolite Analysis Shell) (http://antismash.secondarymetabolites.org/ accessed on 5 December 2021) was applied for the extraction of the probable secondary metabolite gene cluster of the isolate. Mauve software was used (http://gel.ahabs.wisc.edu/mauve accessed on 5 December 2021) for draft genome comparison [61].

### 4.8. Characterization of the Antimicrobial Metabolite(s)

#### 4.8.1. Thin Layer Chromatography (TLC) Analysis

Preliminary separation of the metabolite(s) was performed for both ethyl acetate and dichloromethane extracts using TLC analysis. The crude extracts were spotted on separate Silica TLC coated plates 20 × 20 cm (pre-coated with silica gel 60 F254, Merck, Germany) and developed in three different solvent systems. The three solvent systems were: ethyl acetate: dichloromethane (9:1); ethyl acetate: methanol (9:1); and dichloromethane: methanol (6.5:3.5). The fractionated metabolites were observed under UV light at 254 nm (absorbance) and 365 nm (fluorescence) [62].

#### 4.8.2. Liquid Chromatography–Mass Spectroscopy (LC/MS) Analysis

LC/MS analysis was performed at Center for Drug Discovery Research and Development, Faculty of Pharmacy, Ain Shams University, Cairo, Egypt. The analysis was performed using ESI-MS positive and negative ion acquisition mode with a XEVO TQD triple quadruple instrument, Waters Corporation, Milford, MA01757 USA, mass spectrometer. The stationary phase was a ACQUITY UPLC-BEH C18 1.7 µm-2.1 × 50 mm Column (Santa Clara, CA, USA). The mobile phase was gradient elution consisting of water containing 0.1% formic acid and acetonitrile containing 0.1% formic acid with a flow rate of 0.2 mL/min. 

## 5. Conclusions

In conclusion, *Paenibacillus ehimensis* MZ921932 is a rich source for antimicrobial metabolites production that retains activities against MDR Gram-positive and Gram-negative bacteria as well as *Candia*. The metagenomic nanopore sequence analysis method was a rapid, easy, and efficient method for the preliminary detection of the nature of the expected active metabolites. The LC/MS spectral analysis was very helpful for further confirmation of the nature of the respective active metabolites, such as β-lactone, petrobactin, and macrobrevin. Future studies have to be conducted to purify the active metabolites and confirm their antimicrobial activities, particularly against MDR pathogens. Metagenomics analysis should be studied more for a better understanding of the microbial community and its influence in gene expression.

## Figures and Tables

**Table 1:** Preliminary screening against Gram-positive test organisms.

Isolate Code	Gram Positive Test Organisms					
	SE1	SE2	SE3	VRSA1	VRSA2	VRSA3
SP1	+	+	+	+	+	+
SP2	+	+	+	+	+	+
SP3	-	-	-	-	-	-

+: inhibits growth, -: no inhibition and.SE1, *S. epidermidis* isolate 1; SE2, *S. epidermidis* isolate 2; SE3, *S. epidermidis* isolate 3; VRSA1, vancomycin resistance *S. aureus* isolate 1; VRSA2, vancomycin resistance *S. aureus* isolate 2; VSRA3, vancomycin resistance *S. aureus* isolate 3.

**Table 2:** Preliminary screening against Gram-negative test organisms.

Isolate Code	Gram Negative Test Organisms					
	*E. coli* ATCC 25922	EC1	EC2	KP1	KP2	KP3
SP1	+	+	+	+	+	+
SP2	-	-	-	-	-	-
SP3	-	-	-	±	-	-

+: inhibits growth, -: no inhibition and ±: partial inhibition. EC1, MDR *E. coli* isolate 1; EC2; MDR *E. coli* isolate 2; KP1, MDR *K. pneumoniae* isolate 1; KP2, MDR *K. pneumoniae* isolate 2; KP3, MDR *K. pneumoniae* isolate 3.

**Table 3:** Preliminary screening against tested *Candida* spp.

Isolate Code	*Candida* spp. Test Organisms					
	CA1	CA2	CA3	CS1	CS2	CS3
SP1	+	+	+	+	+	+
SP2	-	-	+	+	+	+
SP3	±	-	-	±	-	-

+: complete inhibition; -: no inhibition; ±: partial inhibition; CA1, *C. albicans* isolate 1; CA2, *C. albicans* isolate 2; CA3, *C. albicans* isolate 3; CS1, *C. auris* isolate 1; CS2, *C. auris* isolate 2; CS3, *C. auris* isolate 3.

**Table 4:** The antimicrobial activity of the solvent extracts of *Paenibacillus ehimensis* isolate MZ921932.

Test Organisms	Mean Zone of Inhibition (mm) ± SD	
	Dichloromethane Extract	Ethyl Acetate Extract
*S. aureus* ATCC 25293	19 ± 1.0	13 ± 0.5
VRSA2	15 ± 0.5	13 ± 1.0
KP1	20 ± 1.0	11 ± 1.0
KP2	14 ± 1.0	14 ± 0.5
EC1	13 ± 0.5	13 ± 0.5
EC2	14 ± 0.5	13 ± 0.5
*C. albicans* ATCC 10231	11 ± 0.5	14 ± 1.0
CA1	11 ± 1.0	-

-, absence of inhibition zone. VRSA2, vancomycin resistance *S. aureus* isolate 2; EC1, MDR *E. coli* isolate 1; EC2; MDR *E. coli* isolate 2; KP1, MDR *K. pneumoniae* isolate 1; KP2, MDR *K. pneumoniae* isolate 2; CA1, *C. albicans* clinical isolate 1.

**Table 5:** Antimicrobial resistance profile of the bacterial clinical isolates.

Gram-Positive		Gram-Negative	
Clinical Isolate Code	Resistance Pattern	Clinical Isolate Code	Resistance Pattern
SE1, SE2, SE3	CLI, CN, FOX, CIP	KP1	AMC, ATM, CTX, CAZ, CRO, FEP, CIP, SXT, TET, IMP, ETP, DOR, CT, PB, FF, RA, CN
VRSA1	VAN, FOX	KP2	AK, AMC, ATM, CTX, CAZ, CRO, FEP, CIP, SXT, TET, IMP, ETP, DOR, CT, FF, RA, CN
VRSA2, VSRA3	VAN, CLI, CN, FOX, CIP	KP3	AK, AMC, ATM, CTX, CAZ, CRO, FEP, CIP, SXT, TET, IMP, ETP, DOR, FF, RA, CN
		EC1	CTX, IMP
		EC2	AK, AMC, ATM, CTX, CAZ, CRO, FEP, CIP, SXT, TET, IMP, ETP, DOR, FF, RA,

Glycopeptides: VAN = vancomycin, Macrolides: CLI = clindamycin Beta-lactams: AMC = Amoxicillin/clavulanic ATM = Aztreonam FOX = cefoxitin CTX = Cefotaxime CAZ = Ceftazidime CRO = Ceftriaxone FEP = Cefepime DOR = Doripenem ETP = Ertapenem IMP = Imipenem; Aminoglycoside: AK = Amikacin CN = Gentamicin Quinolones: CIP = Ciprofloxacin Polymyxins: CT = Colistin PB = Polymyxin B; Sulfonamides/Diaminopyrimidine: SXT = Sulfamethoxazole/Trimethoprim Tetracyclines: TE = Tetracycline; TGC = Tigecycline Phosphonic acid derivative: FF = Fosfomycin Rifamycins: RA = Rifamycin.

## Supplementary Materials

The following are available online at https://www.mdpi.com/article/10.3390/antibiotics11010012/s1, Figure S1. Metagenomics analysis identifying the microbial diversity and their abundance. Figure S2. TLCs of Dichloromethane extract and Ethyl acetate extracts in different mobile phases observed under UV lamp at 365 nm (fluorescence) and 254 nm (absorbance). (a) & (b) ethyl acetate:Methanol (9:1); (c) & (d) Dichloromethane:Methanol (6.5:3.5).
