# Supplementary material for: Exploring the Nature of the Antimicrobial Metabolites Produced by Paenibacillus ehimensis Soil Isolate MZ921932 Using a Metagenomic Nanopore Sequencing Coupled with LC-Mass Analysis
Source: Antibiotics (Basel). 2021 Dec 22;11(1):12. doi: 10.3390/antibiotics11010012 (PMC8773065; doi:10.3390/antibiotics11010012)
Supplement: Supplementary file 1 [file antibiotics-11-00012-s001.zip › antibiotics-1520371-supplementary.pdf]

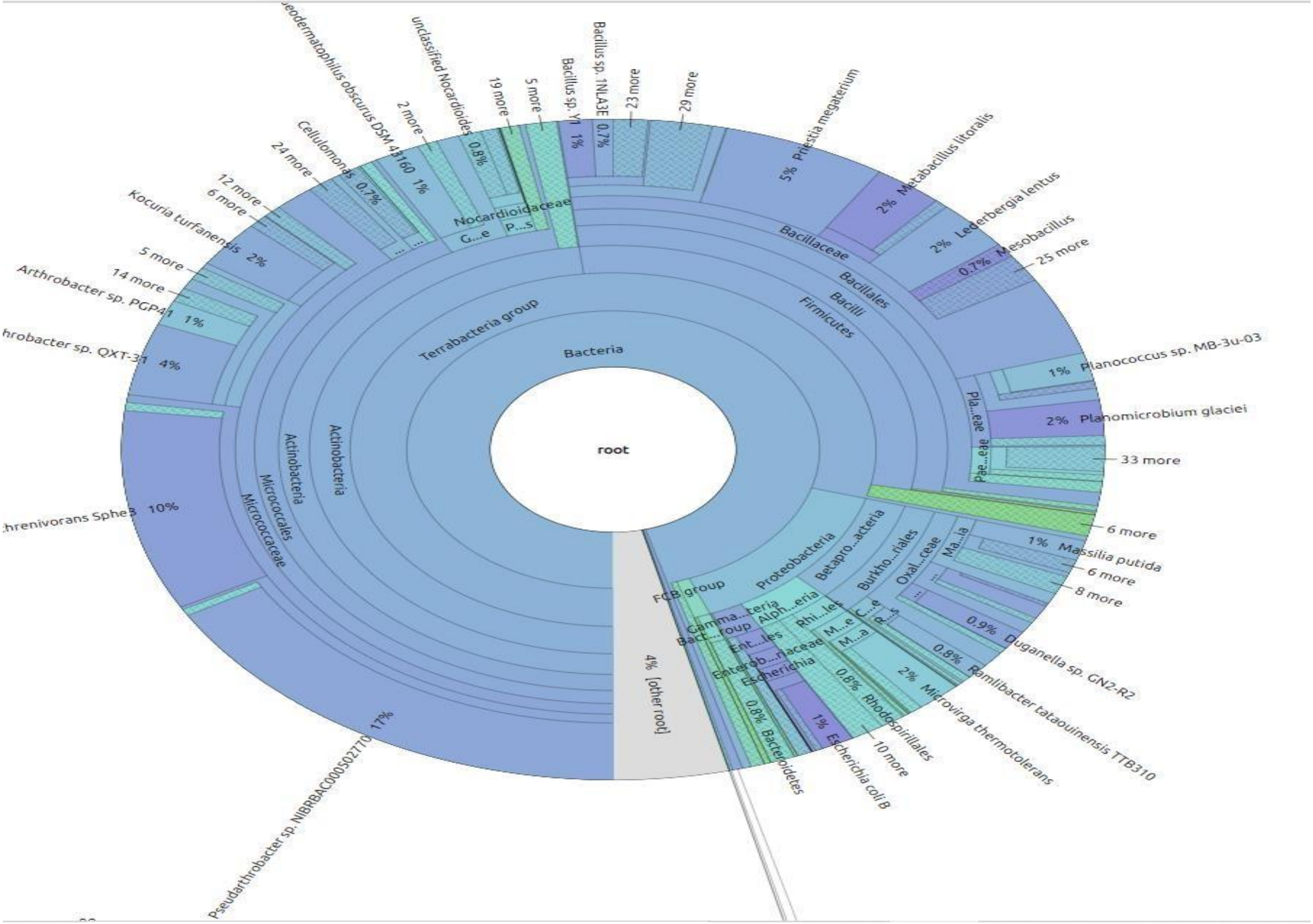

Figure S1. Metagenomics analysis identifying the microbial diversity and their abundance

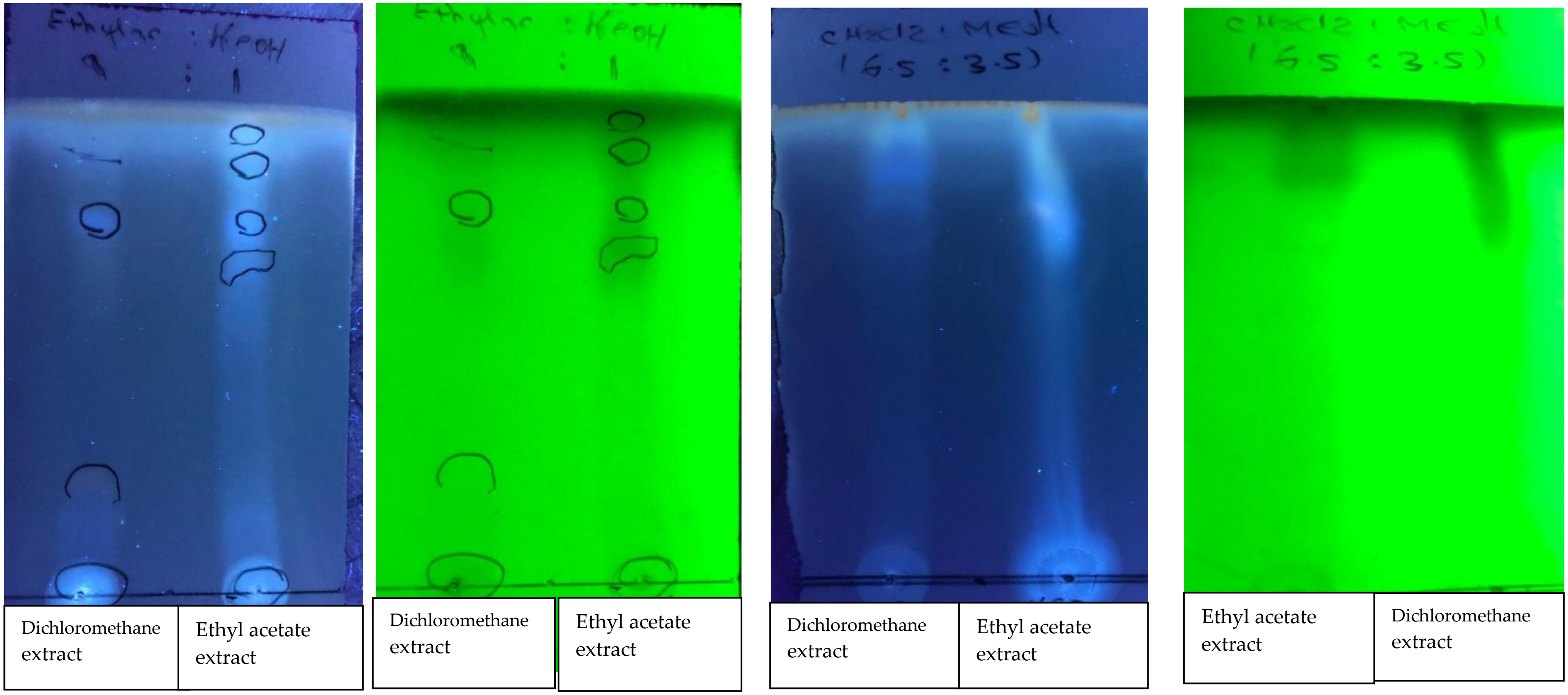

(a) **(b)** **(c)** **(d)**

Figure S2. TLCs of Dichloromethane extract and Ethyl acetate extracts in different mobile phases observed under UV lamp at 365 nm (fluorescence) and 254 nm (absorbance). (a) & (b) ethyl acetate : Methanol (9:1); (c) & (d) Dichloromethane : Methanol (6.5: 3.5)
